# Clearance of Chronic Actinic Dermatitis With Dupilumab Therapy in Chinese Patients: A Case Series

**DOI:** 10.3389/fmed.2022.803692

**Published:** 2022-02-24

**Authors:** Kamran Ali, Liming Wu, HaiYue Lou, Jianbo Zhong, YunMi Qiu, JiaYang Da, JingPeng Shan, KaiNing Lu

**Affiliations:** ^1^Department of Dermatology, International Education College of Zhejiang Chinese Medical University, Hangzhou, China; ^2^Department of Dermatology, Affiliated Hangzhou First People's Hospital, Zhejiang University School of Medicine, Hangzhou, China; ^3^Department of Oncological Surgery, Affiliated Hangzhou First People's Hospital, Zhejiang University School of Medicine, Hangzhou, China

**Keywords:** chronic actinic dermatitis, dupilumab, photo-dermatoses, hydroxychloroquine, photodermatitis

## Abstract

Chronic actinic dermatitis (CAD) is a rare chronic immunological photo-dermatosis resulting in pruritic eczematous eruption on sun-exposed skin to ultraviolet (UV) light. The disease mechanism may include a delay-type hypersensitivity reaction to an endogenous photo-induced antigen, postulated to be UVR-altered DNA, but the exact pathophysiology is unknown. Minimum erythema dosing and patch testing are diagnostic tools of CAD. There are limited safe and effective treatment options for CAD. Herein, a case series of three patients with severe recalcitrant CAD is presented after being treated with dupilumab off-label. The patients in this study had persistent severe disease and taken the first-line management plan, which consists of topical calcineurin inhibitors (TCI), topical corticosteroids (TCS), and strict photoprotection. However, the above treatment options were not able to control the symptoms. The patients were treated with dupilumab 600 mg first dose, 300 mg biweekly subcutaneously (SC), and hydroxychloroquine. Dupilumab showed excellent clinical benefits, including safe and well-tolerated in chronic actinic dermatitis. Further studies are required to be carried out before being applied in clinical practice.

## Introduction

Chronic actinic dermatitis (CAD) was previously known as photosensitive dermatitis, eczema, actinic reticuloid (AR), and immunological-mediated photo-dermatosis. CAD is defined as chronic pruritus eczematous lesions and often lichenified plaques

predominantly upon sun-exposed skin areas in patients above 50 years old, who often have outdoor activities (1). Chronic actinic dermatitis is seen in all skin types, more prevalent in patients of lower skin phenotypes. However, in Fitzpatrick, skin Types V and VI are most likely seen in younger patients (2). There are limited treatment options available for CAD, and it has inadequate therapeutic effects and severe side effects. Dupilumab is an IgG human monoclonal antibody (mAB) that binds to the IL-4 receptor (IL-4R-alpha), inhibits the lL-4 and IL-13 cytokines signaling pathway, and reduces the level of Th2 biomarkers. Dupilumab has promisingly improved skin barrier function and the health-related quality of life HRQoL in the clinical trial for atopic dermatitis (AD). Dupilumab has been approved by the food and drug administration (FDA) for moderate-to-severe AD, and CAD shared the similar Th2-mediated inflammatory pathogenesis as atopic dermatitis (AD) (3, 4). This study presents a case series of patients with CAD treated with **Dupilumab**, Hydroxychloroquine, and topical calcineurin inhibitors (0.3% tacrolimus ointment) to overcome the disease severity. Following treatment showed a significant outcome, and no side effects were observed during the follow-up period.

## Methods

### Patients and Assessments

The patients were clinically diagnosed as chronic actinic dermatitis, and we took the complete medical and family history. Photo testing indicated UVA-MED negative for one patient, and the other two patients showed positive at 24 h, and UVB-MED was positive at 24 h. A complete blood count and the erythrocyte sedimentation rate were normal. Anti-SSA, antinuclear antibodies (ANA), and anti-SSB antibodies were negative. The patients were recalcitrant to topical and systemic therapy and were injected 600 mg initial dose of dupilumab, followed by 300 mg biweekly with the combination of hydroxychloroquine 100 mg BID per oral (PO). Patients continued strict photoprotective measures and topical medications. No additional systematic immunosuppressant therapy was added to the treatment. The dupilumab outcome was observed and recorded based on clinical presentation. Treatment outcomes were recorded as well as any side effects of dupilumab. Follow-up was done biweekly to observe the change in lesion and disease progress. In addition, we extracted the data on the history of CAD treatment and systemic comorbidities from patients' previous medical records. Data of patients with CAD treated with dupilumab and hydroxychloroquine were retrospectively analyzed from February 2021 to October 2021.

## Results

### Case Presentation

**Figure 1:**
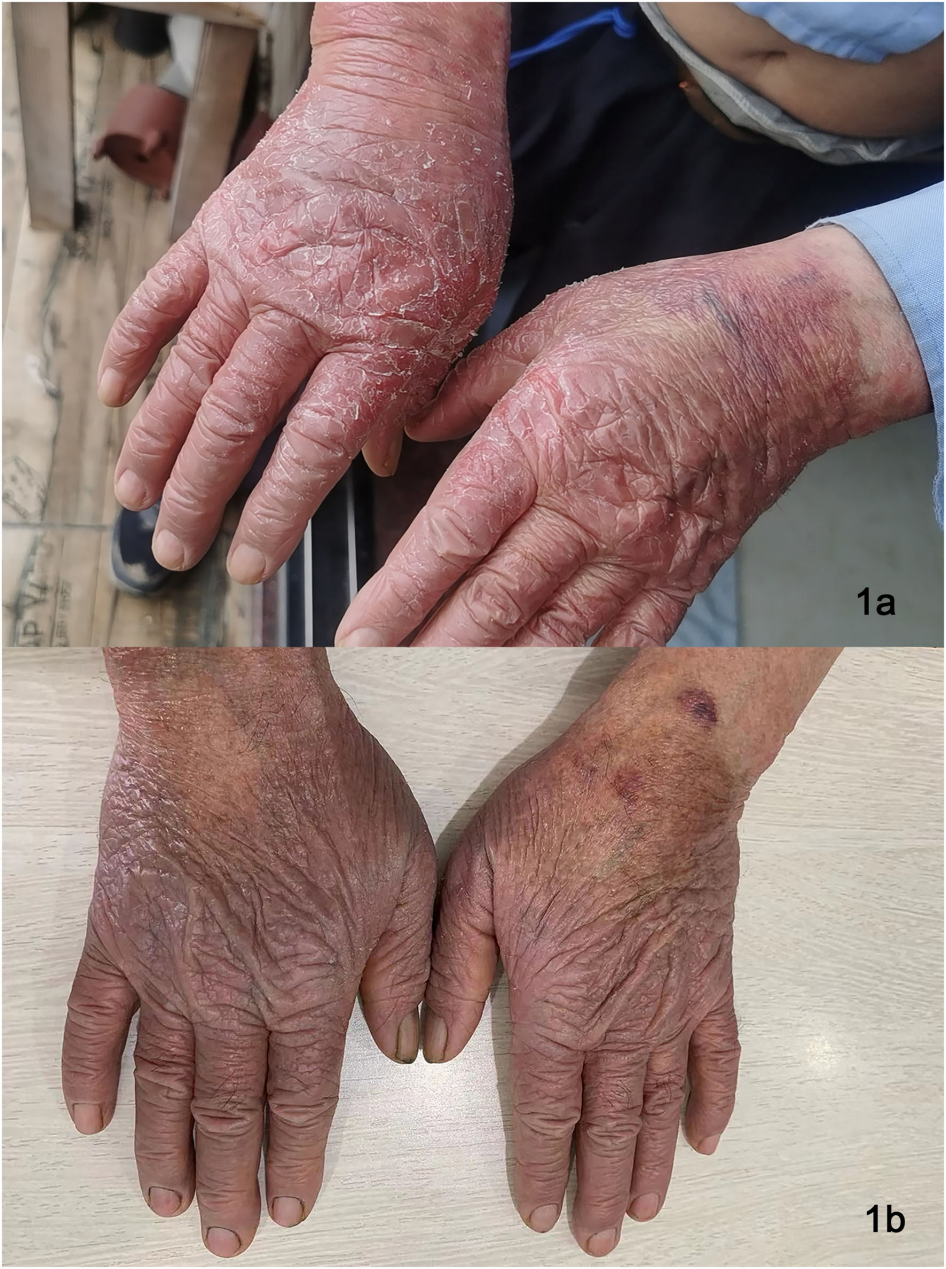
Lesions on dorsa of hands of Patient 2 before dupilumab therapy **(a)**, remarkably improved lesions on dorsal hands after treatment **(b)**.

**Figure 2:**
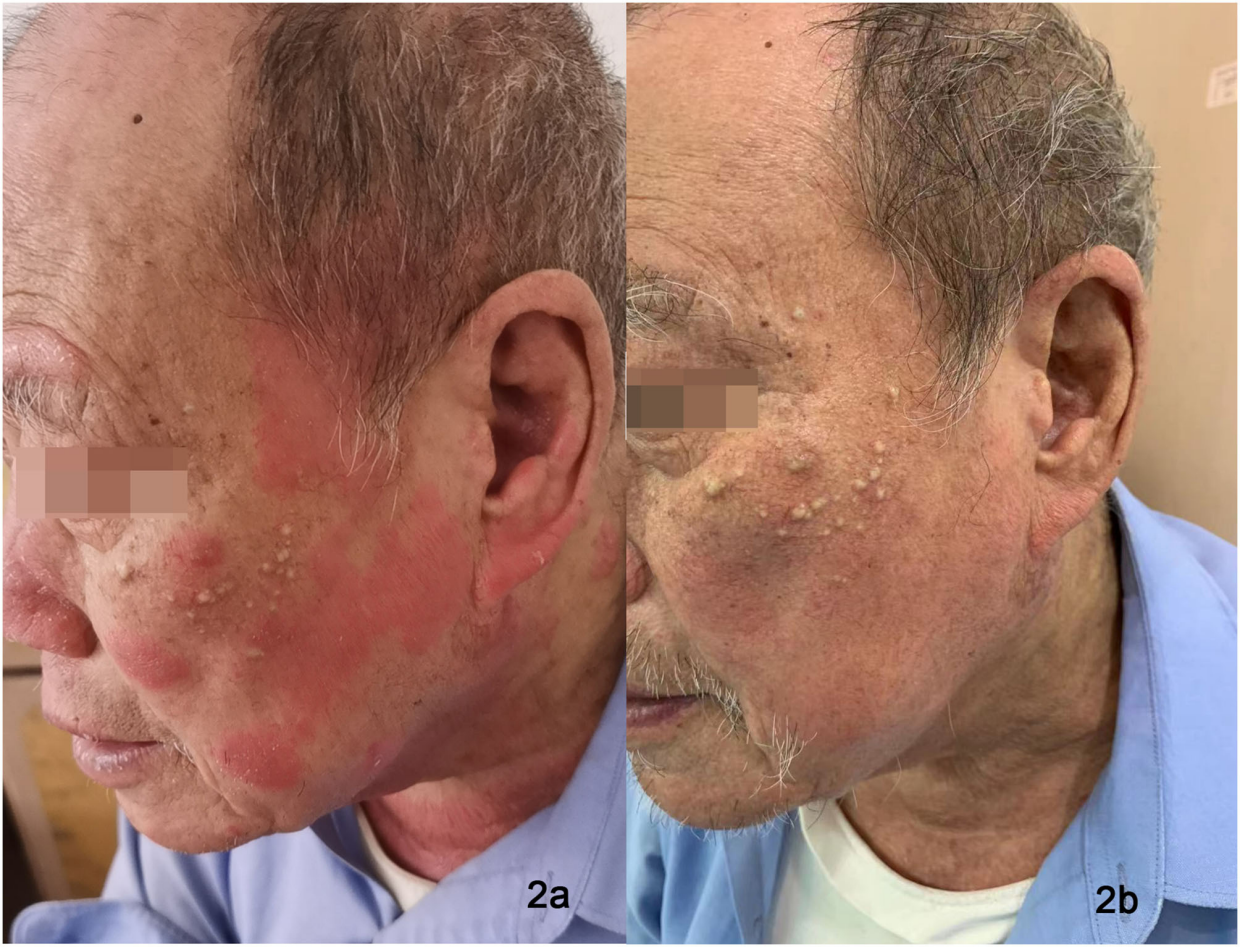
Eczematous, scaly, and excoriated plaques on the face of Patient 2 before treatment **(a)**. Nearly clearance of lesions after dupilumab treatment **(b)**.

Three male patients with CAD were diagnosed clinically and treated in our dermatology department since February, 2021 to October, 2021. The patients had a history of mild chronic eczematous, scaly, and excoriated papules. They presented with pruritic edematous red plaques on the sun-exposed areas, including scalp, forehead, ears, face, and dorsa of hands, but no lesions were observed on the shaded areas of the body (Table 1; Figures 1a, 2a). They have experienced increased symptoms in summer on sun-exposed skin, but less in winter. Upon examination, the clinical presentations were consistent with chronic actinic dermatitis. The patients were diagnosed based on clinical presentation and MED results. One patient had a history of atopic dermatitis (AD). The patients have been treated with topical mometasone furoate and oral antihistamines and diligent photoprotection without significant improvement. Systematic treatment was initiated due to topical therapy's worsening symptoms and failure to photoprotective clothing and sunscreen. Methotrexate, hydroxychloroquine, and azathioprine did not show satisfactory results (Table 1). After discussion with the patients, dupilumab was initiated off-label at a 600-mg loading dose and 300 mg subcutaneously (SC) biweekly with the concomitant of hydroxychloroquine 100 mg two times a day for 8–20 weeks. All other systemic treatments were discontinued. However, strict photoprotection and topical ointment were continued. The efficacy and adverse events were analyzed.

**Table 1:** Patients with chronic actinic dermatitis (CAD) treated with dupilumab (*n* = 3).

	**Patient 1**	**Patient 2**	**Patient 3**
Age	58	77	69
Sex	Male	Male	Male
Duration of CAD (Years)	>10	4	1
Clinical presentation	Well-demarcated Erythematous excoriated lichenified pruritic plaques on sun-exposed areas (face, fore arm, dorsa of hands and scalp), increased symptoms in summer during outdoor activities. Spared shaded areas.	Pruritic edematous excoriated lichenified plaques on sun-exposed areas (face, upper chest, dorsa of hands and scalp), increased symptoms in summer specially during outdoor activities. Shaded areas are spared.	Well-demarcated phot-induced eczematous on sun-exposed areas (face, upper chest, fore arm, dorsa of hands and scalp), increased symptoms in summer during outdoor activities. No lesion were found on the shaded areas of the body.
Serum IgE levels	150 IU/mL (normal 20–200 IU/mL)	88 IU/mL (normal 20–200 IU/mL)	Not done
Severity measurement tools (IGA, EASI, Pruritic-NRS)	IGA: 4^v0^/1^A^ EASI: 10.3^v0^/1^A^ BSA:21^v0^/6^A^ Pruritic-NRS: 7^v0^/2^A^	IGA: 4^v0^/1^A^ EASI: 13^v0^/1.7^A^ BSA:20^v0^/8^A^ Pruritic-NRS: 8^v0^/1^A^	IGA: 4^v0^/2^A^ EASI: 11^v0^/2^A^ BSA:26^v0^/10^A^ Pruritic-NRS: 8^v0^/3^A^
Previous treatment	Corticosteroid (T, PO), TCI, methotrexate, antihistamine, hydroxychloroquine (2)	Corticosteroid (T, PO), TCI, methotrexate, Azathioprine, hydroxychloroquine, mycophenolate mofetil (2)	Corticosteroids (T), TCI, antihistamines and hydroxychloroquine (2)
Photo-testing (MED)	UVA: positive at 7 mJ/cm2 at 24 h (normal range: ≥10 mJ/cm2 ) UVB: positive at 25 mJ/cm2 at 24 h (normal range: ≥40 mJ/cm2 )	UVA: negative at 24 h UVB: positive at 15 mJ/cm2 at 24 h (normal range: ≥40 mJ/cm2)	UVA: positive at 3 mJ/cm2 at 24 h (normal range: ≥10 mJ/cm2 ) UVB: positive at 18 mJ/cm2 at 24 h (normal range: ≥40 mJ/cm2 )
Treatment with Dupilumab	Dupilumab 600 mg, 300 mg biweekly+ hydroxychloroquine	Dupilumab 600 mg, 300 mg biweekly+ hydroxychloroquine	Dupilumab 600 mg, 300 mg biweekly+ hydroxychloroquine
Treatment duration	16 weeks (continued)	20 weeks (continued)	8 weeks (continued)
Follow up	Clearance of lesions	Nearly clearance of lesions.	Significant reduced pruritus and rash
Overall effects	↑*↑↑*	↑*↑↑*	↑↑
Complications	none	none	none
Comments	History of AD Yes	no	no

*PO, per oral; T, topical; TCIs, topical calcineurin inhibitors; MED, minimal erythema dose; V0, visit zero; A, after treatment; overall effects: → , none; ↑, mild; ↑↑, moderate; ↑↑↑, complete*.

All three patients showed reduced lichenified edematous plaques and decreased pruritus after 1 month of treatment with dupilumab and hydroxychloroquine. Patients 1 and three experienced reduced edematous plaques, scaling, erythema, and pruritus associated with the affected area in 4 weeks. The lesions were nearly cleared after 16- week of treatment. No adverse events were observed. Patient two had exacerbations of the erythematous lesion after the initial dose, but edematous flares were cleared with continued treatment with dupilumab for 12 weeks (Figures 1b, 2b); he did not receive any other systemic therapy. Post-treatment photo-testing was not performed or patients' denial to do repeating photo-testing. However, patients resumed their daily activities without photoprotection and did not complain about disease severity and photosensitivity during the treatment. The patients provided informed photo consent before collecting the images. Details are described in Table 1.

## Discussion

The pathogenesis of chronic actinic dermatitis (CAD) remains unknown. Due to a loss of photo-immunosuppression ability, allowing the photo-induced antigen to activate the hypersensitive reaction, it is more likely to be delayed-type hypercreativity reaction, the more rigorously photo-allergic reaction in allergic contact dermatitis (ACD). These allergens develop when photo-induction (UV-radiation) causes molecular changes in patients with CAD, and some research has suggested that these allergens are driven by or related to UVR-altered DNA (5, 6).

Management of chronic actinic dermatitis and photo-dermatoses is involved in strict sun protection, photoprotective clothing, UVA, and UVB protective sunscreen (physical or sunscreen) but also includes topical and systemic corticosteroids and PUVA therapy. Narrowband ultraviolet B (NB UVB) phototherapy has shown remarkable therapeutic effects and a safety profile in Chinese patients (7). Azathioprine, cyclosporine, mycophenolate mofetil, and hydroxyurea are the steroid-sparing agents that have efficacy in CAD. Long-term use of these drugs has significant health-related risks and side effects; so new safe and effective treatment options are necessary (1, 2). The above case series demonstrated the comprehensive therapeutic option for chronic actinic dermatitis with dupilumab and hydroxychloroquine. None of our patients reported any adverse effects.

Furthermore, in the literature review, we identify three reports with a total of 10 patients (8–10). All the patients showed a moderate-to-complete reduction in disease severity. Two patients developed conjunctivitis and were successfully treated with ophthalmic corticosteroids (20% of the cases) (8). Only one patient had to suspend the treatment with dupilumab because of the DFR (dupilumab facial redness). About 10% of the total reported cases (10). Dupilumab is an IgG4 human mAB that binds to the IL-4 receptor (IL-Rα), inhibiting the transmission of the interleukin (IL-4) and interleukin (IL-13) cytokines signaling. Dupilumab is the first systemic biological agent to treat moderate to severe AD.

In previous studies, it was found that 85 percent of patients with atopic dermatitis (AD) treated with dupilumab had a significant reduction in the Eczema Area and Severity Index score (EASI) when compared to patients who received a placebo (35%) (2, 11). It is challenging to speculate the precise mechanism of action through which dupilumab improved the disease severity in patients with CAD. However, durable and long-lasting responses reveal that dupilumab targets vital inflammatory pathways of atopic dermatitis (AD) and other Th2 cell-mediated inflammatory skin diseases, such as CAD, As Ko et al. have recently suggested that T cells may play a role in the dysbalance of Th1/Th2 in CAD, in atopic dermatitis, there is an over-activation of the Th2 in the acute stage. However, the chronic phase is manifested by Th1 immune response and sustained Th2 -cells activation (12, 13). According to the study, UVB-induced CD3+, CD4+, and CD8^−^ regulatory T cells suppress the immune system through the release of the immune-regulatory cytokines IL-4 and IL-10 (14). Stephen E. Ullrich et al. demonstrated that UV irritation suppresses the antigen-presenting capacity to T helper-1 T (Th1) cells, whereas antigen presentation to T helper-2 type T (Th2) cells is enhanced (4). Despite the fact that the immunological pathways of atopic dermatitis and chronic actinic dermatitis are not the same, however, there is overlapping. Russell SC, et al. first reported seven young British patients with AD, who were photosensitive (15). Subsequently, Creamer D, et al. and Ogboli MI, et al. also reported CAD in young sufferers with a history of atopic dermatitis (16, 17). Nevertheless, an extensive long-term study would be necessary to determine the direct efficacy of this drug on CAD and reveal the similar inflammatory nature of CAD and AD. In the pieces of literature mentioned above, 3 out of 10 patients (30%) had a history of AD.

Hydroxychloroquine belongs to the 4-aminoquinoline derivatives type antimalarial drug, which has antiproliferative, anti-inflammatory, photoprotective, and immunomodulatory effects. It inhibits UV-induced reactions, effectively alleviates the rash, and plays an excellent role in sun protection (18). Our patients were treated with hydroxychloroquine alone or with other immunosuppression, but the results were unsatisfactory. It is suggested that dupilumab inhibits the IL-4 and IL-13 transmission and may interact with the CAD pathogenic cascade, potentially implicating Th2 in the pathophysiology of CAD. However, hydroxychloroquine has photoprotective effects, and it can inhibit UV-induced reactions, which have an important role in CAD pathogeneses. We speculated that the concomitant dupilumab and hydroxychloroquine increase the primary outcome of disease control in patients with CAD.

In conclusion, the treatment of chronic actinic dermatitis is complicated, and there are no standard and safe therapeutic options. Our case study is small; meanwhile, we presented this case series to provide the clinician with another potential therapeutic option to treat CAD. Hence, dupilumab and hydroxychloroquine showed excellent outcomes in our study, and combination therapy could be considered in managing compliant CAD. Further studies are needed to carry out to use in clinical practice.

## Conclusion

Chronic actinic dermatitis treatment is complicated, and currently available therapeutic options have limited efficacy and safety concerns. Dupilumab has shown significant improvement in patients with CAD in our study. Further clinical trials are needed to confirm the long-term safety and efficacy of dupilumab in CAD before adding it to a standard treatment option.

## Data Availability Statement

The raw data supporting the conclusions of this article will be made available by the authors, without undue reservation.

## Ethics Statement

Ethical review and approval was not required for the study on human participants in accordance with the local legislation and institutional requirements. The patients/participants provided their written informed consent to participate in this study. Written informed consent was obtained from the individual(s) for the publication of any potentially identifiable images or data included in this article.

## Author Contributions

KA: wrote the original paper. LW: edit and correct the paper. HL: collect case data. JZ and JS: literature search. YQ: language editing. JD: images editing. KL: provided article ideas and article review. All authors contributed to the article and approved the submitted version.

## Conflict of Interest

The authors declare that the research was conducted in the absence of any commercial or financial relationships that could be construed as a potential conflict of interest.

## Publisher's Note

All claims expressed in this article are solely those of the authors and do not necessarily represent those of their affiliated organizations, or those of the publisher, the editors and the reviewers. Any product that may be evaluated in this article, or claim that may be made by its manufacturer, is not guaranteed or endorsed by the publisher.
